# Incidence and predictors of difficult nasotracheal intubation with airway scope

**DOI:** 10.1007/s00540-013-1778-2

**Published:** 2014-01-17

**Authors:** Koyu Ono, Tomoko Goto, Daishi Nakai, Shuhei Ueki, Seiichiro Takenaka, Tomomi Moriya

**Affiliations:** 1 Departments of Dentistry and Maxillofacial Surgery, Itoh Dento-Maxillofacial Hospital, 4-14 Kokaihonmachi, Kumamoto, 860-0851 Japan; 2 Departments of Anesthesiology, Itoh Dento-Maxillofacial Hospital, Kumamoto, Japan

**Keywords:** Airway scope, Video laryngoscope, Difficult nasotracheal intubation

## Abstract

**Purpose:**

The airway scope (AWS) improves views of the larynx during orotracheal intubation. However, the role of the AWS in routine nasotracheal intubation has not been studied adequately.

**Methods:**

One hundred and three patients undergoing dental and maxillofacial surgery that required general anesthesia and nasotracheal intubation were enrolled. The study was approved by our Institution Review Board, and written informed consent was obtained from all patients. We evaluated the success rate of AWS intubation and the incidence of difficult nasotracheal intubation using a modified intubation difficulty scale (IDS) to examine preoperative characteristics and intubation profiles. Categories were difficult intubation (IDS ≥5), mildly difficult (IDS = 1–4), and intubation without difficulty (IDS = 0). We also assessed the incidence of the use of Magill forceps or cuff inflation (the cuff of endotracheal tube is inflated with 10–15 ml air) to guide the endotracheal tube into the glottis.

**Results:**

AWS nasotracheal intubation was 100 % successful. The cuff inflation technique was used in 37 patients. Neither Magill forceps nor other devices were needed for any patient during AWS use. The incidence of difficult nasotracheal intubation was 10 % (*n* = 10). Of the patients, 61 % (*n* = 63) had mildly difficult intubation and 29 % (*n* = 30) had no difficulty. Patients with difficult intubation were more likely to be male and to have a larger tongue and a higher Cormack grade than in the other two groups. Complications, involving minor soft tissue injury, were observed in only 1 patient (1 %).

**Conclusion:**

The AWS achieves a high success rate for nasotracheal intubation with cuff inflation in patients undergoing dental and maxillofacial surgery.

## Introduction

Nasotracheal intubation usually is accomplished using direct laryngoscopes such as the Machintosh or McCoy. Although direct laryngoscopes provide a sightline view of the airway during nasotracheal intubation, the patient’s neck must be extended and Magill forceps are needed to guide the endotracheal tube into the glottis. The Airway Scope (AWS; Hoya, Tokyo, Japan), a video laryngoscope designed for oral tracheal intubation, has proven useful for patients with difficult airways [1, 2], such as restricted neck movement [3–5]. Another recent study also demonstrated that the AWS offers better intubation conditions than a Macintosh laryngoscope during nasotracheal intubation [6]. Nevertheless, inserting an endotracheal tube with AWS may occasionally fail, despite good visualization of the glottis [7]. In addition, nasotracheal intubation sometimes is needed to guide the tip of endotracheal tube into the glottis with use of Magill forceps or cuff inflation technique. Reports on a small number of patients found the cuff inflation technique useful for guiding the tip of nasotracheal tube into the glottis under AWS or Airtraq [8, 9]. However, the role of AWS in nasotracheal intubation with guidance with either Magill forceps or cuff inflation has not been studied adequately. We assessed the success rate of AWS intubation and the incidence of difficult nasotracheal intubation in patients who underwent dental and maxillofacial surgery. We also analyzed the predictors for difficult intubation and the role of cuff inflation technique on guiding the endotracheal tube to the glottis during nasotracheal intubation by AWS.

## Methods

### Study population

The Institutional Research Ethics Committee of Itoh Dento-Maxillofacial Hospital approved the study protocol, and written informed consent was obtained from each patient. A total of 103 patients (aged 16 years and older) undergoing elective dental maxillofacial surgery were enrolled between December 2011 and April 2012. Exclusion criteria were ASA physical status III–IV, age <16 years, and limited mouth opening (<3 cm). Data of patient characteristics and preanesthetic risk were evaluated by a single attending anesthesiologist. Parameters and common predictors of difficult intubation included age, gender, body mass index (BMI), snoring, sleep apnea syndrome (history of diagnosed, treated, or episode of apnea), mandibular retrusion (posterior displacement of the mandibula), restricted neck movement (limited extension, limited flexion), large tongue (disproportionately large size occupying oropharyngeal space with teeth press marks on the lateral borders of the tongue), Mallampati grade, and upper lip bite. The upper lip bite test class was graded according the following criteria: class I, lower incisors can bite the upper lip above the vermilion line; class II, lower incisors can bite the upper lip below the vermilion line; and class III, lower incisors cannot bite the upper lip [10].

### Patient management

AWS was performed by five dental anesthesiology residents with more than 3 months of experience and were supervised by one attending anesthesiologist. When the patients arrived in the operating room, they were monitored by electrocardiogram, noninvasive blood pressure, pulse oximetry, and capnometry (20, 30, 40, 50, 60 s step-up alarm of apnea set available). Each patient’s head was positioned on a dental chair support to achieve a neutral position. After oxygen was administered for at least 3 min with a face mask, anesthesia was induced with IV fentanyl 100 μg, propofol 2 mg/kg, and sevoflurane. Appropriate neuromuscular blockade was achieved with rocuronium 0.6 mg/kg before airway manipulation.

Procedural scores for intubation difficulty were evaluated during tracheal intubation, as well as the modified Cormack–Lehane grade [11], position of the AWS top blade in vallecula or epiglottis, number of attempts, dependent need for external manipulation or cuff inflation technique, Magill forceps, a gum elastic bougie, or a bronchofiber. When the tip of the endotracheal tube was withdrawn into the laryngopharynx, the cuff of the tube was sequentially inflated with 10–15 ml air (cuff inflation technique) until guiding the glottis and then was deflated after the tip of the tube entered into laryngeal inlet. The trachea was intubated with a Parker Flex-Tip endotracheal tube (Parker Medical, Ranch, CO, USA) or Ivory nasal tube (Smith Medical, Hythe, UK). The anesthesiologist assigned to the case chose the internal diameter size of the endotracheal tube based on the patient’s characteristics and surgical procedure. If surgery was expected to be longer than 2 h, a softer Ivory nasal tube was selected to prevent nasal injury. A failed intubation was defined as an attempt that required more than 30 s. When nasal intubation failed, the endotracheal tube was connected to an anesthetic circuit and the lungs were manually ventilated with the patient’s mouth occluded by hand. All complications of hypoxia [oxygen saturation measured by pulse oximetry (SpO_2_) <90 %] during nasotracheal intubation, and dental, pharyngeal, tracheal, or laryngeal injury were recorded.

### Intubation difficulty sale

Difficulty of intubation was assessed according to the modified intubation difficulty scale (IDS) developed by Adnet et al. [12] on the basis of seven variables: N1, number of additional intubation attempts; N2, number of additional operators; N3, number of alternative intubation techniques used such as bronchofiber; N4, AWS view as defined by modified Cormack and Lehane (0 = grade 1, the vocal cords were completely visible; 1 = grade 2a, partial view of the vocal cords; 2 = grade 2b, only the arytenoids and epiglottis seen; 3 = grade 3, only the epiglottis visible); N5, lifting force required during AWS (0 = normal, 1 = increased); N6, need to apply external laryngeal pressure to improve glottis (0 = not applied, 1 = external laryngeal pressure was used); and N7, aid technique in intubation (0 = not applied, 1 = lifting head, 2 = use of cuff inflation, 3 = use of Magill forceps or gum elastic bougie). The IDS score was the sum of N1 through N7. We defined three groups of patients according to the IDS values: not difficult (IDS score = 0), mildly difficult (IDS score = 1–4), and difficult (IDS score ≥5).

### Statistical analysis

Data are reported as mean (± SD) and incidences (both absolute and percentage). One-way analysis of variance was used to compare data for parametric data between groups. When statistical significance was found, post hoc comparisons were made by Bonferroni’s method. The chi-square statistic test or Fisher’s exact test with Bonferroni correction for multiple comparisons was used to compare all nonparametric data between groups. A *P* value <0.05 was considered significant.

## Results

Nasotracheal intubation using the AWS was successful in all 103 patients. Tracheal intubation was judged easy in 30 patients (29 %), mildly difficult in 63 patients (61 %), and difficult in 10 patients (10 %). Preoperative and other characteristics for all patients are summarized in Table 1. More patients with difficult intubation were male and had large tongues and a higher Cormack grade than the other two groups. There were no statistically significant differences for other variables among the three groups. There were no incidences of hypoxia during intubation in any group.

**Table 1:** Patient characteristics and preoperative intubation conditions

	Not difficult (*n* = 30)	Mildly difficult (*n* = 63)	Difficult (*n* = 10)	*P* value
Age, years (mean ± standard deviation)	35 ± 15.5	32 ± 14.2	355 ± 15.8	0.665
Gender (male/female)	4/26	24/39	6/4^a^	0.029
Height (m)	1.6 ± 0.06	1.6 ± 0.08	1.7 ± 0.09^a^	0.006
Weight (kg)	53.1 ± 9.4	56.4 ± 10.7	61.7 ± 13.0	0.771
Body mass index (kg/m^2^)	20.9 ± 2.80	21.2 ± 2.68	21.7 ± 2.64	0.706
Snoring (%)	7 (23.3)	18 (28.6)	4 (40)	0.825
Sleep apnea syndrome (%)	1 (3.3)	1 (1.6)	2 (2)	0.150
Mandibular retrusion (%)	3 (10)	5 (7.9)	1 (10)	0.913
Restricted neck movement (%)	0 (0)	0 (0)	1 (10)	0.395
Large tongue (%)	1 (3.3)	9 (14.3)	5 (50)^b^	0.008
Mallampati grade I/II/III/IV	20/10/0/0	42/18/3/0	8/2/0/0	0.944
Upper lip bite I/II/III	30/0/0	60/3/0	9/1/0	0.935

^a^Significantly different from “not difficult” by post hoc analysis

^b^Significantly different from “not difficult” and “mildly difficult” by post hoc analysis

The number of nasotracheal intubation attempts and optimization maneuvers required were significantly higher in patients with difficult intubation compared with the other two groups (Table 2). Cuff inflation was needed to guide the endotracheal tube to the visualized glottis in 28 (44 %) patients of the mildly difficult group and to 9 (90 %) patients of the difficult group. No Magill forceps or other devices were needed for any patient. Complications were noted in only 1 patient (1 %), that involving minor soft tissue injury. Before surgery began, the surgeon checked the oral cavity for bleeding and found a submucosal hematoma of the left palatal arch in a 32-year-old man undergoing sagittal split ramus osteotomy. After confirming that there was no bleeding from the soft palate, the endotracheal tube was removed. No major complications, such as dental, pharyngeal, tracheal, or laryngeal injury were found.

**Table 2:** Intubation profiles for nasotracheal intubation difficulty

	Not difficult (*n* = 30)	Mildly difficult (*n* = 63)	Difficult (*n* = 10)	*P* value
Modified Cormack–Lehane grade (I/IIa/IIb/III)	30/0/0/0	50/13/0/0	4/5/1/0^a^	0.038
Number of attempts (1/2/3)	30/0/0	58/4/1	1/7/2	<0.001
Additional management of intubation (none/lifting head/cuff inflation/Magill’s forceps)	30/0/0/0	5/30/28/0	0/1/9/0	<0.001
Lifting force	0	7	9	<0.001
External laryngeal manipulation	0	6	9	<0.001
Tracheal tube (Ivory tube/Parker tube)	16/14	32/31	7/3	0.721
Position of AWS top (Vallecula/Epiglottis)	9/21	13/50	2/8	0.754

*AWS* airway scope

^a^Significantly different from “not difficult” by post hoc analysis

## Discussion

Our results have shown that the AWS achieved a high rate of success for nasotracheal intubation with cuff inflation in patients who underwent dental and maxillofacial surgery. Males and those with large tongues and higher Cormack grade were more common among the patients with difficult nasotracheal intubation.

Previous studies have demonstrated that the AWS reduces the difficulty of tracheal intubation in restricted cervical patients and in a manikin with tongue edema [3, 4, 13]. On the other hand, our study demonstrated that the AWS increased the need for additional attempts and optimization maneuvers in men and patients with large tongues. In this study, failed intubation was defined as procedures longer than 30 s in which the accumulated number of intubation attempts has increased. A large tongue may make inserting INTLOCK (single-use blade) of the AWS along the palate to be difficult. Abdallah et al. [14] reported that a narrowed oropharyngeal space in obese patients might impede manipulation of the relatively bulky INTLOCK of the AWS. In addition, male gender and snoring are risk factors for obstructive sleep apnea [15, 16]. Although snoring was not a risk factor for difficult nasal intubation in this study, further work is needed on whether the AWS improves the grade of glottis views in patients with obstructive sleep apnea syndrome.

Magill forceps sometimes are needed to guide the tip of the endotracheal tube into the glottis during nasotracheal intubation. Several studies have reported that cuff inflation or a gum elastic bougie is useful to guide the tip of tube located posterior toward the glottis [8, 9, 17]. We used cuff inflation in 37 patients. Cuff inflation is a simple and useful way to correct the position of the endotracheal tube tip during nasotracheal intubation by the AWS. Although we did not use the gum elastic bougie in this study, this device may be required in patients when cuff inflation fails. In our institution, the AWS has reduced the use of bronchofiber-guide intubation and changed our airway management of nasotracheal intubation in patients undergoing dental and maxillofacial surgery.

Complications involved one male patient with minor submucosal hematoma of his palatal arch. He had a large tongue and required two intubation attempts. His large tongue may have disturbed the insertion of the INTLOCK of the AWS along the palate. The AWS blade may be too large for the patient with a narrow oropharyngeal space. Ogino et al. [18] reported on a small female patient who developed distinct airway edema after palatal laceration caused by inserting the AWS. Generally, we use a single size for the INTLOCK component of the AWS. Recently, smaller sizes of the INTLOCK component have been released as thin types for adults, children, and infants. Because of the relatively bulky blade, the AWS should be inserted carefully along the palate in patients with narrow oropharyngeal space such as caused by a large tongue.

Our study has some limitations. First, it was performed in a single institution. Thus, our institutional standards or patients might have biased the results. Second, we modified the definition of difficult intubation to be an IDS score of 5 or more, which often differs between institutions.

In conclusions, the AWS achieved a high rate of success for nasotracheal intubation with cuff inflation in patients undergoing dental and maxillofacial surgery. A minor complication, minor soft tissue injury, was noted in one patient. There were no major complications. The AWS should be inserted carefully along the palate in patients with oropharyngeal spaces made narrow by a large tongue or other anatomic factors.
